# Supplementary material for: The landscape of fear conceptual framework: definition and review of current applications and misuses
Source: PeerJ. 2017 Sep 12;5:e3772. doi: 10.7717/peerj.3772 (PMC5600181; doi:10.7717/peerj.3772)
Supplement: Appendix S1 [file peerj-05-3772-s001.docx]

**APPENDIX I: THE HISTORICAL BASE LEADING TO**

**THE LOFS;**

**Trophic cascades**

The world is green because predators control the populations of grazing species concluded

Hairston (Hairston, Smith & Slobodkin, 1960) in what later became known as the ``Green

World Hypothesis''. Since this ground-breaking paper, a large focus in community ecology

was directed at the study of trophic cascades, i.e., the idea that through direct predation the

size of a population impacts multiple trophic levels below it and the size of the population

of prey dictates the availability of resources that sustain the predators above them.

The textbook example used to teach these interactions is population fluxes in Lotka_

Volterra of lynx-hare (and mastings) predator_prey cycles in Canadian boreal forests

(Hewitt, 1921; Fox & Bryant, 1984; Krebs et al., 1995; Lima, 1998). This example highlights

the trophic cascades from a bottom-up perspective, i.e., how the availability of resources

influences the populations of predators. Resource availability dictates the availability of

niches for species to occupy in the community (Vincent & Brown, 2005). However, the

top-down interactions greatly dictate the traits the species must have to survive within

those niches. Robert Paine pioneered the study of the predation implications on prey when

he described how a diverse predator community results in diverse prey community (Paine,

1963). In this example, the predation pressure from multiple intertidal zone predators

(sea stars) removed the competitive ability of the dominant barnacles and allowed for

higher mollusk diversity. More recently, Schmitz & Price (2011) showed a strong positive

correlation between the biomass of arachnid predators on the vegetation biomass in an

agricultural system. In this system, spiders feed on grasshoppers that damage vegetation. The

biomass of spiders thus positively correlates with the health of the vegetation crop. These

examples show that the predators negatively affect the prey populations and thus indirectly

have positive effects on the vegetation. However, consumptive-effects did not entirely

explain how predators control prey (and vice-versa). Thus a study of non-consumptive

predator effects was created, named the ``ecology of fear'' by Joel Brown (Brown, Laundré & Gurung, 1999), with the aim to answer two major questions: (1) Do predators control

the populations of prey solely by consuming them? And (2) would the evolution of prey

species to manage the risk of predation not overcome the negative impacts these predators

have on their prey populations?

**Non- consumptive effects of predators: an “Ecology of Fear”**

One does not have to study wildlife behavior to understand the effects predation risk has

on animal behavior. All one has to do, is think of our own body's response to a risky

situation. Our bodies instinctively respond to the risk in the environment by producing

stress hormones. This production results in increased blood pressure, pulse and sensory

sensitivity. In essence, our bodies tell us to get out of harm's way as fast as we can. In

this example of our own day-to-day life we find the evidence for the millions of years

evolutionary race between predators and prey to maintain the energetic needs of both

groups. Prey evolve mechanisms to avoid the risk, and predators find ways to out-gun

these defense mechanisms.

In non-human examples we expect that predators would have to find a balance

between over-hunting a naïve food resource into extinction and starvation from aiming

to hunt highly vigilant prey (Brown & Vincent, 1992; Lima, 1998; Lima, 2002; Brown,

Laundré & Gurung, 1999; Wolf & Mangel, 2007). The evolutionary arms-race between the

predators and their prey results in predators managing the fear of the prey into an optimal

state of vigilance by limiting their encounter rates (Embar, Mukherjee & Kotler, 2014).

Overstimulating the perception of risk in the prey, would lower the hunting success of

the predator to unsustainable levels. Meanwhile, the prey species evolved to counteract

the predator management through a variety of behavioural strategies, or choices. I will

examine a few case studies of such strategies.

Many species choose their habitat based on the risk which this habitat poses to them. For

example, heteromyid rodents choose habitat based on the density of vegetation. Species

(kangaroo rats) that can hop out of harm's way prefer the un-encumbered open, and

species that are limited in predator evasion strategies (pocket mice) find shelter under

thick vegetation (Rosenzweig, 1973). In kangaroo rats specifically the presence of vipers was

shown to be a driver of the choice of the open habitat (Bouskila, 1995). In another example,

Gerbilline rodents responded to owls with clear preference for sheltered microhabitats

(Kotler, Blaustein & Brown, 1992; Abramsky et al., 1996; Abramsky, Rosenzweig & Subach,

1997; Kotler et al., 2002; St. Juliana et al., 2011; Embar et al., 2014). Habitat fragmentation,

or edge effect, has been shown to affect the habitat use (predominantly in the form of

avoidance) by songbirds (Storch, Woitke & Krieger, 2005; Fischer & Lindenmayer, 2007).

Larger animals (ungulates and primates) have been shown to select habitat where the

sightlines allow good visibility of approaching predators (Tadesse, 2012; Abu Baker

& Brown, 2013; Coleman & Hill, 2014). Lastly, a number of studies studying wolf-elk

interactions show that based on habitat variability the strategies applied by the elk to avoid

predation (vigilance, habitat selection) can vary greatly (Hebblewhite, Merrill & McDonald,

2005; Eisenberg et al., 2014).

Other strategic choices of prey driven by the predator-prey dynamics can include

dietary selection and movement patterns. For example (again in the Heteromyid rodents),

foragers better equipped for risk management (kangaroo rats) have a more diverse diet

than the foragers less well equipped for predator management (pocket mice) who forage

what they can (Davidson, Brown & Inouye, 1980). In another example, in the Simpson

Desert, dasyurid marsupials avoid risk by covering large distances to search for refuge.

These small mammals (20 grams on average) inhabit burrows in the swale of sand dunes

located away from the resource dense habitats at the dune crests (Haythornthwaite, 2005;

Haythornthwaite & Dickman, 2006). All of these adaptations clearly suggest that a trade-off

between resources and predation risk occurs within a spatial dynamic, and as such these

predator-prey games can and should be studied using a spatial analysis, a ``landscape of

fear'' (LOF).

This conceptual framework was expanded to study the effects of landscape heterogeneity

(Brown & Kotler, 2004) culminating in measured maps combining vegetation, refuge,

resource availability and risk. In an example with cape ground squirrels (*Xerus inauris*),

the LOF was interpreted as a cost benefit analysis of energetic values over change in

the landscape (Joules/ meter) (Van Der Merwe & Brown, 2008). This conversion allowed

researchers to weigh the study of energetic gain from food patches and compare those gains

to the cost of risk avoidance (cf. Brown, 1988)

Literature Cited:

Abramsky Z., Rosenzweig ML., Subach A. 1997. Gerbils under threat of owl predation : isoclines and isodars. Oikos 78:81–90.

Abramsky Z., Strauss E., Subach A., Riechman A., Kotler BP. 1996. The effect of barn owls (Tyto alba) on the activity and microhabitat selection of Gerbillus allenbyi and G. pyramidum. Oecologia 105:313–319. DOI: 10.1007/BF00328733.

Abu Baker M., Brown JS. 2013. Foraging and habitat use of common duikers , Sylvicapra grimmia , in a heterogeneous environment within the Soutpansberg, South Africa. African Journal of Ecology 52:1–10.

Bouskila A. 1995. Interactions between predation risk and competition : a field study of kangaroo rats and snakes. Ecology 76:165–178.

Brown JS. 1988. Patch use as an indicator of habitat preference, predation risk, and competition. Behavioral Ecology and Sociobiology 22:37–47. DOI: 10.1007/BF00395696.

Brown JS., Kotler BP. 2004. Hazardous duty pay and the foraging cost of predation. Ecology Letters 7:999–1014. DOI: 10.1111/j.1461-0248.2004.00661.x.

Brown JS., Laundre JW., Gurung M. 1999. The ecology of fear: optimal foraging, game theory, and trophic interactions. Journal of Mammalogy 80:385–399.

Brown JS., Vincent TL. 1992. Organization of predator-prey communities as an evolutionary game. Evolution 46:1269–1283.

Coleman BT., Hill RA. 2014. Living in a landscape of fear: the impact of predation, resource availability and habitat structure on primate range use. Animal Behaviour 88:165–173. DOI: 10.1016/j.anbehav.2013.11.027.

Davidson DW., Brown JH., Inouye RS. 1980. Competition and the structure of granivore communities. BioScience 30:233–238.

Eisenberg C., Hibbs DE., Ripple WJ., Salwasser H. 2014. Context dependence of elk ( Cervus elaphus ) vigilance and wolf ( Canis lupus ) predation risk. Canadian Journal of Zoology 92:727–736. DOI: 10.1139/cjz-2014-0049.

Embar K., Mukherjee S., Kotler BP. 2014. What do predators really want ? The role of gerbil energetic state in determining prey choice by Barn Owls. Ecology 95:280–285.

Embar K., Raveh A., Hoffmann I., Kotler BP. 2014. Predator facilitation or interference: a game of vipers and owls. Oecologia 174:1301–9. DOI: 10.1007/s00442-013-2760-2.

Fischer J., Lindenmayer DB. 2007. Landscape modification and habitat fragmentation : a synthesis. Global Ecology and Biogeography 16:265–280. DOI: 10.1111/j.1466-8238.2006.00287.x.

Fox JF., Bryant JP. 1984. Instability of the snowshoe hare and woody plant interaction. Oecologia 63:128–135. DOI: 10.1007/BF00379794.

Hairston NG., Smith FE., Slobodkin LB. 1960. Community structure , population control, and competition. American Naturalist 94:421–425. DOI: 10.1086/521238.

Haythornthwaite AS. 2005. Microhabitat use and foraging behaviour of Sminthopsis youngsoni (Marsupialia : Dasyuridae) in arid central Australia. Wildlife Research 32:609–615. DOI: 10.1071/WR04126.

Haythornthwaite AS., Dickman CR. 2006. Long-distance movements by a small carnivorous marsupial: How Sminthopsis youngsoni (Marsupialia: Dasyuridae) uses habitat in an Australian sandridge desert. Journal of Zoology 270:543–549. DOI: 10.1111/j.1469-7998.2006.00186.x.

Hebblewhite M., Merrill EH., McDonald TL. 2005. Spatial Decomposition of Predation Risk Using Resource Selection Functions : An Example in a Wolf-Elk Predator-Prey System. Oikos 111:101–111.

Hewitt CG. 1921. In The Conservation of the Wild Life of Canada. New York, New York: Charles Scribner’s Sons.

St. Juliana J., Kotler BP., Brown JS., Mukherjee S., Bouskila A. 2011. The foraging response of gerbils to a gradient of owl numbers. Evolutionary Ecology Research 13:869–878.

Kotler BP., Blaustein L., Brown JS. 1992. Predator Facilitation: the combined effect of snakes and owls on the foraging behavior of gerbils. Annals of Zoology Fennici 29:199–206.

Kotler BP., Brown JS., Dall SRX., Gresser S., Ganey D., Bouskila A. 2002. Foraging games between gerbils and their predators : temporal dynamics of resource depletion and apprehension in gerbils. Evolutionary Ecology Research 4:495–518.

Krebs CJ., Boutin S., Boonstra R., Sinclair a R., Smith JN., Dale MR., Martin K., Turkington R. 1995. Impact of food and predation on the snowshoe hare cycle. Science 269:1112–1115. DOI: 10.1126/science.269.5227.1112.

Lima SL. 1998. Nonlethal effects in the ecology of predator-prey interactions. BioScience 48:25–34. DOI: 10.2307/1313225.

Lima SL. 2002. Putting predators back into behavioral predator–prey interactions. Trends in Ecology & Evolution 17:70–75. DOI: 10.1016/S0169-5347(01)02393-X.

Van Der Merwe M., Brown JS. 2008. Mapping the landscape of fear of the cape ground squirrel (Xerus inauris). Journal of Mammalogy 89:1162–1169. DOI: 10.1644/08-MAMM-A-035.1.

Paine RT. 1963. Food web complexity and species diversity. The American Naturalist 100:65–75. DOI: 10.2307/2678832.

Rosenzweig ML. 1973. Habitat selection experiments with a pair of coexisting Heteromyid rodent species. Ecology 54:111–117.

Schmitz OJ., Price JR. 2011. Convergence of trophic interaction strengths in grassland food webs through metabolic scaling of herbivore biomass. Journal of Animal Ecology 80:1330–1336. DOI: 10.1111/j.1365-2656.2011.01882.x.

Storch I., Woitke E., Krieger S. 2005. Landscape-scale edge effect in predation risk in forest-farmland mosaics of central europe. Landscape Ecology 20:927–940. DOI: 10.1007/s10980-005-7005-2.

Tadesse S. 2012. Habitat quality and foraging ecology of Mountain Nyala (Tragelaphus buxtoni) in the Munessa Forest and the Bale Mountains National Park, South-Eastern Ethiopia. Ben Gurion University of the Negev.

Vincent TL., Brown JS. 2005. Evolutionary game theory , natural selection , and Darwinian dynamics. Cambridge, UK: Cambridge University Press.

Wolf N., Mangel M. 2007. Strategy , compromise , and cheating in predator – prey games. Evolutionary Ecology Research 9:1293–1304.
