# Supplementary material for: The landscape of fear conceptual framework: definition and review of current applications and misuses
Source: PeerJ. 2017 Sep 12;5:e3772. doi: 10.7717/peerj.3772 (PMC5600181; doi:10.7717/peerj.3772)
Supplement: Appendix S2 [file peerj-05-3772-s002.docx]

**APPENDIX II: BIBLIOGRAPHY OF MANUSCRIPTS**

**REVIEWED FOR THIS ANALYSIS**

Abu Baker MA., Brown JS. 2010. Islands of fear: effects of wooded patches on habitat suitability of the striped mouse in a South African grassland. Functional Ecology 24:1313–1322. DOI: 10.1111/j.1365-2435.2010.01757.x.

Abu Baker M a., Brown JS. 2011. Variation of within-day foraging costs in the striped mouse (*Rhabdomys pumilio*). Mammalian Biology 76:654–656. DOI: 10.1016/j.mambio.2011.02.005.

Abu Baker M., Brown JS. 2013. Foraging and habitat use of common duikers , *Sylvicapra grimmia* , in a heterogeneous environment within the Soutpansberg, South Africa. African Journal of Ecology 52:1–10.

Altendorf KB., Laundré JW., Lopez Gonzalez CA., Brown JS., González CAL., Brown JS. 2001. Assessing effects of predation risk on foraging behavior of mule deer. Journal of Mammalogy 82:430–439.

Arias-Del Razo I., Hernández L., Laundré JWJ., Velasco-Vázquez L., Hernández L., Laundré JWJ. 2012. The landscape of fear: habitat use by a predator (*Canis latrans*) and its main prey (*Lepus californicus and Sylvilagus audubonii*). Canadian Journal of Zoology 90:683–693. DOI: 10.1139/Z2012-036.

Atkins A., Redpath SM., Little RM., Amar A. 2017. Experimentally Manipulating the Landscape of Fear to Manage Problem Animals. The Journal of Wildlife Management 81:610–616. DOI: 10.1002/jwmg.21227.

Bedoya-Perez MA., Carthey AJR., Mella VSA., McArthur C., Banks PB. 2013. A practical guide to avoid giving up on giving-up densities. Behavioral Ecology and Sociobiology 67:1541–1553. DOI: 10.1007/s00265-013-1609-3.

Benhaiem S., Delon M., Lourtet B., Cargnelutti B., Aulagnier S., Hewison AM., Morellet N., Verheyden H. 2008. Hunting increases vigilance levels in roe deer and modifies feeding site selection. Animal Behaviour 76:611–618. DOI: 10.1016/j.anbehav.2008.03.012.

Beschta RL., Ripple WJ. 2013. Are wolves saving Yellowstone’s aspen? A landscape-level test of a behaviorally mediated trophic cascade : comment. Ecology 94:1420–1425.

Blanchard P., Lauzeral C., Chamille-Jammes S., Yoccoz NG., Pontier D. 2016. Analyzing the proximity to cover in a landscape of fear: a new approach applied to fine-scale habitat use by rabbits facing feral cat predation on Kerguelen archipelago. PeerJ 1:1–16.

Bleicher SS. 2014. Divergent behaviour amid convergent evolution: common garden experiments with desert rodents and vipers. University of Illinois at Chicago.

Breviglieri CPB., Piccoli GCO., Uieda W., Romero GQ. 2013. Predation-risk effects of predator identity on the foraging behaviors of frugivorous bats. Oecologia 173:905–912. DOI: 10.1007/s00442-013-2677-9.

Brook LA., Johnson CN., Ritchie EG. 2012. Effects of predator control on behaviour of an apex predator and indirect consequences for mesopredator suppression. Journal of Applied Ecology 49:1278–1286. DOI: 10.1111/j.1365-2664.2012.02207.x.

Brown JS. 2010. Ecology of fear. In: Breed MD, Moore J eds. Encyclopedia of Animal Behaviour. Oxford: Elsevier Ltd, 581–587.

Brown JS., Kotler BP. 2004. Hazardous duty pay and the foraging cost of predation. Ecology Letters 7:999–1014. DOI: 10.1111/j.1461-0248.2004.00661.x.

Ciuti S., Muhly TB., Paton DG., McDevitt AD., Musiani M., Boyce MS. 2012a. Human selection of elk behavioural traits in a landscape of fear. Proceedings. Biological sciences / The Royal Society 279:4407–4416. DOI: 10.1098/rspb.2012.1483.

Ciuti S., Northrup JM., Muhly TB., Simi S., Musiani M., Pitt JA., Boyce MS. 2012b. Effects of Humans on Behaviour of Wildlife Exceed Those of Natural Predators in a Landscape of Fear. PLoS ONE: 1-6. DOI: 10.1371/journal.pone.0050611.

Coleman BT., Hill RA. 2014. Living in a landscape of fear: the impact of predation, resource availability and habitat structure on primate range use. Animal Behaviour 88:165–173. DOI: 10.1016/j.anbehav.2013.11.027.

Cromsigt JPGM., Kuijper DPJ., Adam M., Beschta RL., Churski M., Eycott A., Kerley GIH., Mysterud A., Schmidt K., West K. 2013. Hunting for fear: innovating management of human-wildlife conflicts. Journal of Applied Ecology 50:544–549. DOI: 10.1111/1365-2664.12076.

Druce DJ., Brown JS., Kerley GIH., Kotler BP., Mackey RL., Slotow R. 2009. Spatial and temporal scaling in habitat utilization by klipspringers ( *Oreotragus oreotragus* ) determined using giving-up densities. Austral Ecology 34:577–587. DOI: 10.1111/j.1442-9993.2009.01963.x.

Dupuch A., Morris DW., Ale SB., Wilson DJ., Moore DE. 2014. Landscapes of fear or competition? Predation did not alter habitat choice by Arctic rodents. Oecologia 174:403–412. DOI: 10.1007/s00442-013-2792-7.

Dupuch A., Morris DW., Halliday WD. 2013. Patch use and vigilance by sympatric lemmings in predator and competitor-driven landscapes of fear. Behavioral Ecology and Sociobiology 68:299–308. DOI: 10.1007/s00265-013-1645-z.

Eccard J a., Liesenjohann T. 2014. The importance of predation risk and missed opportunity costs for context-dependent foraging patterns. PLoS ONE 9:1–5. DOI: 10.1371/journal.pone.0094107.

Embar K., Kotler BP., Mukherjee S. 2011. Risk management in optimal foragers: the effect of sightlines and predator type on patch use, time allocation, and vigilance in gerbils. Oikos 120:1657–1666. DOI: 10.1111/j.1600-0706.2011.19278.x.

Emerson SE., Brown JS., Linden JD. 2011. Identifying Sykes’ monkeys’, *Cercopithecus albogularis erythrarchus*, axes of fear through patch use. Animal Behaviour 81:455–462. DOI: 10.1016/j.anbehav.2010.11.018.

Gaillard J-M., Hebblewhite M., Loison A., Fuller M., Powell R., Basille M., Van Moorter B. 2010. Habitat-performance relationships: finding the right metric at a given spatial scale. Philosophical Transactions of the Royal Society of London. Series B, Biological sciences 365:2255–2265. DOI: 10.1098/rstb.2010.0085.

Gallagher AJ., Creel S., Wilson RP., Cooke SJ. 2017. Energy Landscapes and the Landscape of Fear. Trends in Ecology & Evolution 32:88–96. DOI: 10.1016/j.tree.2016.10.010.

Gervasi V., Sand H., Zimmermann B., Mattisson J., Linnell JDC., Gervasi V., Sand H., Zimmermann B., Mattisson J., Wabakken P., Linnell JDC. 2013. Decomposing risk: Landscape structure and wolf behavior generate different predation patterns in two sympatric ungulates. Ecological Applications 23:1722–1734.

Gil MA., Zill J., Ponciano JM. 2017. Context- ­ dependent landscape of fear: algal density elicits risky herbivory in a coral reef. Ecology 98:534–544. DOI: 10.1002/ecy.1668.

Halofsky JS., Ripple WJ. 2008. Fine-scale predation risk on elk after wolf reintroduction in Yellowstone National Park, USA. Oecologia 155:869–877. DOI: 10.1007/s00442-007-0956-z.

Hammerschlag N., Broderick AC., Coker JW., Coyne MS., Dodd M., Frick MG., Godfrey MH., Godley BJ., Griffin DB., Hartog K., Murphy SR., Murphy TM., Rose Nelson E., Williams KL., Witt MJ., Hawkes LA. 2015. Evaluating the landscape of fear between apex predatory sharks and mobile sea turtles across a large dynamic seascape. Ecology 96:2117–2126. DOI: 10.1890/14-2113.1.

Heithaus MR., Frid A., Wirsing AJ., Dill LM., Fourqurean JW., Burkholder D., Thomson J., Bejder L. 2007. State-dependent risk-taking by green sea turtles mediates top-down effects of tiger shark intimidation in a marine ecosystem. Journal of Animal Ecology 76:837–844. DOI: 10.1111/j.1365-2656.2007.01260.x.

Hernández L., Laundré JW. 2005. Foraging in the “landscape of fear” and its implications for habitat use and diet quality of elk *Cervus elaphus* and bison *Bison bison*. Wildlife Biology 11:215–220. DOI: 10.2981/0909-6396(2005)11[215:FITLOF]2.0.CO;2.

Iribarren C., Kotler BP. 2012. Foraging patterns of habitat use reveal landscape of fear of Nubian ibex *Capra nubiana*. Wildlife Biology 18:194–201. DOI: 10.2981/11-041.

Jaatinen K., Seltmann MW., Öst M. 2014. Context-dependent stress responses and their connections to fitness in a landscape of fear. Journal of Zoology 294:147–153. DOI: 10.1111/jzo.12169.

Jacob J., Brown JS. 2000. Microhabitat Use , Giving-Up Densities and Temporal Activity as Short- and Long-Term Anti-Predator Behaviors in Common Voles. Oikos 91:131–138.

Kauffman MJ., Brodie JF., Jules ES., Url S. 2013. Are wolves saving Yellowstone’s aspens? A landscape-level test of a behaviorally mediated trophic cascade. Ecology 91:2742–2755. DOI: 10.1890/09-1949.1.

Kauffman MJ., Varley N., Smith DW., Stahler DR., MacNulty DR., Boyce MS. 2007. Landscape heterogeneity shapes predation in a newly restored predator-prey system. Ecology Letters 10:690–700. DOI: 10.1111/j.1461-0248.2007.01059.x.

Kuijper DPJ., de Kleine C., Churski M., van Hooft P., Bubnicki J., Jedrzejewska B. 2013. Landscape of fear in Europe: Wolves affect spatial patterns of ungulate browsing in Białowieza Primeval Primeval Forest, Poland. Ecography 36:1263–1275. DOI: 10.1111/j.1600-0587.2013.00266.x.

Laundré JW. 2010. Behavioral response races , predator — prey shell games , ecology of fear , and patch use of pumas and their ungulate prey. Ecology 91:2995–3007.

Laundré JW., Calderas JMM., Hernández L. 2009. Foraging in the landscape of fear , the predator’s dilemma: Where should I hunt ? The Open Ecology Journal 2:1–6.

Laundré JW., Hernández L., Altendorf KB. 2001. Wolves, elk, and bison: reestablishing the “landscape of fear” in Yellowstone National Park, U.S.A. Canadian Journal of Zoology 79:1401–1409. DOI: 10.1139/cjz-79-8-1401.

Laundre JW., Hernandez L., Lopez Medina P., Campanella A., Lopez-Portillo J., Gonzolez-Romero A., Grajales-Tam KM., Burke AM., Gronemeyer P., Browning DM. 2017. The landscape of fear: the missing link to understand top-down and bottom-up controls of prey abundance ? Ecology 95:1141–1152. DOI: 10.1890/13-1083.1.

Laundre JW., Hernandez L., Ripple WJ. 2010. The landscape of fear: Ecological implications of being afraid. The Open Ecology Journal 3:1–7. DOI: 10.2174/1874213001003030001.

Lone K., Loe LE., Gobakken T., Linnell JDC., Odden J., Remmen JJ., Mysterud A. 2014. Living and dying in a multi-predator landscape of fear: Roe deer are squeezed by contrasting pattern of predation risk imposed by lynx and humans. Oikos 123:641–651. DOI: 10.1111/j.1600-0706.2013.00938.x.

Lyly MS., Villers A., Koivisto E., Helle P., Ollila T., Korpim??ki E. 2015. Avian top predator and the landscape of fear: Responses of mammalian mesopredators to risk imposed by the golden eagle. Ecology and Evolution 5: 503-212 DOI: 10.1002/ece3.1370.

Madin EMP., Madin JS., Booth DJ. 2011. Landscape of fear visible from space. Scientific Reports 1:1–4. DOI: 10.1038/srep00014.

Makin DF., Payne HFP., Kerley GIH., Shrader AM. 2012. Foraging in a 3-D world: how does predation risk affect space use of vervet monkeys? Journal of Mammalogy 93:422–428. DOI: 10.1644/11-MAMM-A-115.1.

Manning AD., Gordon IJ., Ripple WJ. 2009. Restoring landscapes of fear with wolves in the Scottish Highlands. Biological Conservation 142:2314–2321. DOI: 10.1016/j.biocon.2009.05.007.

Massey J., Cubaynes S., Coulson T. 2013. Will central Wyoming elk stop migrating to Yellowstone , and should we care ? Ecology 94:1271–1274.

Matassa CM., Trussell GC. 2011. Landscape of fear influences the relative importance of consumptive and nonconsumptive predator effects. Ecology 92:2258–2266. DOI: 10.1890/11-0424.1.

Mella VSA., Banks PB., Mcarthur C. 2014. Negotiating multiple cues of predation risk in a landscape of fear: What scares free-ranging brushtail possums? Journal of Zoology 294:22–30. DOI: 10.1111/jzo.12146.

Van Der Merwe M., Brown JS. 2008. Mapping the landscape of fear of the cape ground squirrel (*Xerus inauris*). Journal of Mammalogy 89:1162–1169. DOI: 10.1644/08-MAMM-A-035.1.

Nowak K., Le Roux A., Richards SA., Scheijen CPJ., Hill RA. 2014. Human observers impact habituated samango monkeys’ perceived landscape of fear. Behavioral Ecology 25:1–6. DOI: 10.1093/beheco/aru110.

Oriol-Cotterill A., Valeix M., Frank LG., Riginos C., Macdonald DW. 2015. Landscapes of coexistence for terrestrial carnivores: The ecological consequences of being downgraded from ultimate to penultimate predator by humans. Oikos 124:1263–1273. DOI: 10.1111/oik.02224.

Riginos C. 2015. Climate and the landscape of fear in an African savanna. Journal of Animal Ecology 84:124–133. DOI: 10.1111/1365-2656.12262.

Riginos C., Grace JB. 2008. Savanna tree density, herbivores, and the herbaceous community: bottom-up vs. top-down effects. Ecology 89:2228–38.

Ripple WJ., Beschta RL. 2003. Wolf reintroduction, predation risk, and cottonwood recovery in Yellowstone National Park. Forest Ecology and Management 184:299–313. DOI: 10.1016/S0378-1127(03)00154-3.

Ripple WJ., Beschta RL. 2004. Wolves and the Ecology of Fear: Can Predation Risk Structure Ecosystems? BioScience 54:755–766. DOI: 10.1641/0006-3568(2004)054[0755:WATEOF]2.0.CO;2.

Ripple WJ., Beschta RL. 2012. Trophic cascades in Yellowstone: The first 15years after wolf reintroduction. Biological Conservation 145:205–213. DOI: 10.1016/j.biocon.2011.11.005.

Ritchie EG., Johnson CN. 2009. Predator interactions , mesopredator release and biodiversity conservation. Ecology Letters 12:982–998. DOI: 10.1111/j.1461-0248.2009.01347.x.

Rösner S., Mussard-Forster E., Lorenc T., Müller J. 2014. Recreation shapes a “landscape of fear” for a threatened forest bird species in Central Europe. Landscape Ecology 29:55–66. DOI: 10.1007/s10980-013-9964-z.

Rypstra AL., Schmidt JM., Reif BD., Devito J., Matthew H., Rypstra AL., Schmidt JM., Reif BD., Devito J., Persons MH. 2007. Tradeoffs Involved in Site Selection and Foraging in a Wolf Spider : Effects of Substrate Structure and Predation Risk. Oikos 116:853–863.

Schmidt K., Kuijper DPJ. 2015. A “death trap” in the landscape of fear. Mammal Research 60:275–284. DOI: 10.1007/s13364-015-0229-x.

Searle KR., Stokes CJ., Gordon IJ. 2008. When foraging and fear meet: Using foraging hierarchies to inform assessments of landscapes of fear. Behavioral Ecology 19:475–482. DOI: 10.1093/beheco/arn004.

Shrader AM., Brown JS., Kerley GIH., Kotler BP. 2008. Do free-ranging domestic goats show “landscapes of fear”? Patch use in response to habitat features and predator cues. Journal of Arid Environments 72:1811–1819. DOI: 10.1016/j.jaridenv.2008.05.004.

Steele MA., Rompré G., Stratford JA., Zhang H., Suchocki M., Marino S. 2015. Scatterhoarding rodents favor higher predation risks for cache sites: The potential for predators to influence the seed dispersal process. Integrative Zoology 10:257–266. DOI: 10.1111/1749-4877.12134.

Sundararaj V., Mclaren BE., Morris DW., Goyal SP. 2012. Can rare positive interactions become commo carnivores consume livestock? Ecology 93:272–280.

Suraci JP., Clinchy M., Dill LM., Roberts D., Zanette LY. 2016. Fear of large carnivores causes a trophic cascade. Nature Communications 7: 1-5. DOI: 10.1038/ncomms10698.

Swanson A., Arnold T., Kosmala M., Packer C. 2016. In the absence of a “ landscape of fear ”: How lions , hyenas , and cheetahs coexist. Ecology and Evolution 6:8534–8545. DOI: 10.1002/ece3.2569.

Tadesse S. 2012. Habitat quality and foraging ecology of Mountain Nyala (*Tragelaphus buxtoni*) in the Munessa Forest and the Bale Mountains National Park, South-Eastern Ethiopia. Ben Gurion University of the Negev.

Tadesse S., Kotler BP. 2011. Seasonal Habitat Use by Nuian Ibex (*Capra nubiana*) evaluated with behavioral indicators. Israel Journal of Ecology and Evolution 57:223–246.

Themb’alilhlwa AMM., Monadjem A., McCleery R., Belmain SR. 2017. Domestic cats and dogs create a landscape of fear for pest rodents around rural homesteads. PloS ONE 1:1–9. DOI: 10.1371/journal.pone.0171593.

Tolon V., Dray S., Loison A., Zeileis a., Fischer C., Baubet E. 2009. Responding to spatial and temporal variations in predation risk: space use of a game species in a changing landscape of fear. Canadian Journal of Zoology 87:1129–1137. DOI: 10.1139/Z09-101.

Valeix AM., Loveridge AJ., Davidson Z., Murindagomo F., Macdonald DW., Ecology S., Jan N., Chamaill S., Lyon U De., Mendel BG. 2009. Behavioral Adjustments of African Herbivores to Predation Risk by Lions : Spatiotemporal Variations Influence Habitat Use. Ecology 90:23–30.

Willems EP., Hill RA. 2009. Predator-Specific Landscapes of Fear and Resource Distribution: Effects on Spatial Range Use. Ecology 90:546–555.

Zanette LY., Sih A. 2015. Gordon Research Conference on Predator–Prey Interactions: from Genes, to Ecosystems to Human Mental Health. Bulletin of the Ecological Society of America 96:165–173. DOI: 10.1634/stemcells.2005-0336.

Zanette LY., White AF., Allen MC., Clinchy M. 2011. Percived predation risk reduces the number of offspring songbirds produce per year. Science 334:1398–1402.
